# Comparison of the Design of 3-Pole BLDC Actuators/Motors with a Rotor Based on a Single Permanent Magnet

**DOI:** 10.3390/s22103759

**Published:** 2022-05-15

**Authors:** Krzysztof Smółka, Anna Firych-Nowacka, Sławomir Wiak

**Affiliations:** Institute of Mechatronics and Information Systems, Lodz University of Technology, Stefanowskiego 18/22 St., 90-537 Lodz, Poland; anna.firych-nowacka@p.lodz.pl (A.F.-N.); slawomir.wiak@p.lodz.pl (S.W.)

**Keywords:** PM BLDC, motor, actuator, finite element methods, modeling and simulation

## Abstract

Permanent Magnet (PM) Brushless Direct Current (BLDC) actuators/motors have many advantages over conventional machines, including high efficiency, easy controllability over a wide range of operating speeds, etc. There are many prototypes for such motors; some of them have a very complicated construction, and this ensures their high efficiency. However, in the case of household appliances, the most important thing is simplicity, and, thus, the lowest price of the design and production. This article presents a comparison of computer models of different design solutions for a small PM BLDC motor that uses a rotor in the form of a single ferrite magnet. The analyses were performed by using the finite element method. This paper presents unique self-defined parts of basic PM BLDC actuators. With their help, various design solutions were compared with the PM BLDC motor used in household appliances. The authors proved that the reference device is the lightest one and has a lower cogging torque compared to other actuators, but also has a slightly lower driving torque.

## 1. Introduction

Solutions used in household appliances are a frequent element analyzed in the literature (References [1,2,3,4,5,6], for example). One of the important elements of these devices is the electric micromotor. Until a few years ago, household appliances such as washing machines, dishwashers or dryers have been mainly powered by universal motors or squirrel cage induction motors [7,8]. Traditional electric machines used in household appliances have many disadvantages; for instance, they have low energy efficiency and a low starting torque, and there is a problem with sparking brushes and the need to replace them. Nowadays, conventional AC and DC motors are being replaced with new solutions [9]. One of the examples of solutions is Brushless DC Motors, which has become the preferred type of motor in recent years, among others, due to their high efficiency. Additionally, these are usually motors that do not have rotor position sensors. Such solutions are used in some applications where precise position control is not required. The advantages of sensorless solutions are, above all, lower cost, greater reliability and lower complexity of the device. These devices have greater resistance to interference and lower maintenance requirements [10]. Apart from pump applications in household appliances [11], such motors can be found in hard drives [12].

In this study, different types of three-pole BLDC motors were compared with a single-magnet rotor. An existing sensorless BLDC motor used in household appliances as a pump drive was used as the reference motor, for example in dishwashers. The modeling uses Comsol Multiphysics software and the built-in Comsol Parts tool to enable the rapid generation of analog engine designs. The analysis concerns the comparison of drive torques, and cogging torques as a function of the rotor position and as a function of the supplied voltage for various structures. The analysis was made to answer the question of whether the existing motor can be improved by changing the stator structure, without changing the most important parameters of the motor.

## 2. BLDC Actuators/Motors

A Permanent Magnet Brushless DC Motor, PM BLDC motor, is a type of electric motor in which, instead of a mechanical commutator with brushes, an electrically controlled commutator is used, the coils are stationary and the magnets are on the rotor. PM BLDC motors have a lot of advantages over conventional machines, including high efficiency, easy controllability over a wide range of operating speeds, reduced noise, high power density and relatively small dimensions [13]. These actuators are also characterized by a relatively simple design, low moment of inertia, good torque/speed curve and good dynamic properties. Moreover, they are also very reliable [14]. For these reasons, these motors are now widely used in industrial applications [15], automotive applications [16,17,18] and household products [19].

In PM BLDC motors, the role of a mechanical commutator is performed by an electronic system, called an electronic commutator, which generates a control signal depending on the position of the rotor in relation to the windings. These motors are generally controlled by using a three-phase power semiconductor bridge [20].

The position of the rotor relative to the stator is required when the PM BLDC motor is running [21]. The control of PM BLDC motors can be performed in sensor or sensorless mode. In the case of sensor solutions, optical encoders, electromagnetic resolvers, magnetic encoders and hall-effect sensors are used [22]. The main advantage of the sensorless PM BLDC motor control is that the sensor part can be omitted and, thus, the overall cost can be significantly reduced. The disadvantages of sensorless control are the higher requirements for the control algorithms and more complex electronics [23]. The sensorless control methods used in motors for household appliances can be divided into two basic categories: saliency-based sensorless control methods and model-based sensorless control methods [24]. There are many methods of rotor flux position detecting, but the most common methods are based on the derivation of back electromotive force (EMF) signals.

## 3. Models

**Figure 1:**
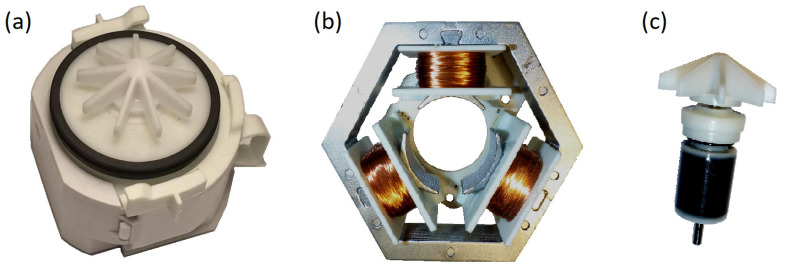
BLDC actuator/motor used in household appliances (54 V, 30 W, 3300 r/min): (**a**) pomp, (**b**) stator and (**c**) rotor.

The article presents various solutions for a sensorless motor with three stator teeth and a simpler rotor based on a single permanent magnet in the form of a ring. This design ensures that it is a simple and cheap solution. All models are related to the real actuator/motor used in dishwasher pumps (Figure 1).

**Figure 2:**
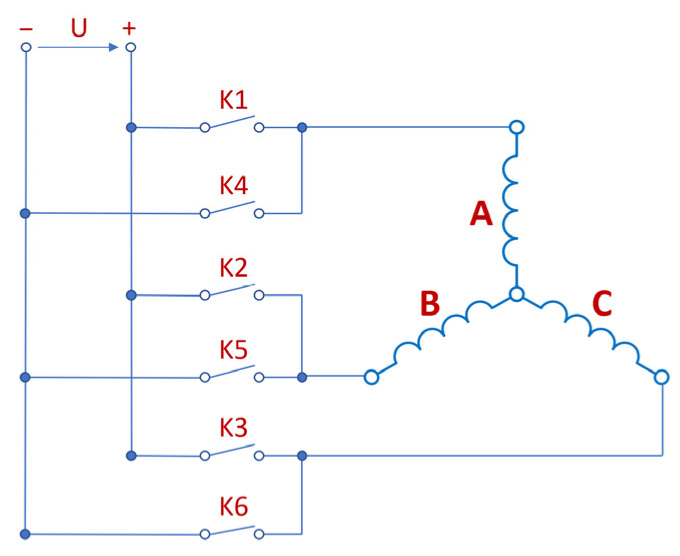
Electrical diagram of the motor with control keys.

**Figure 3:**
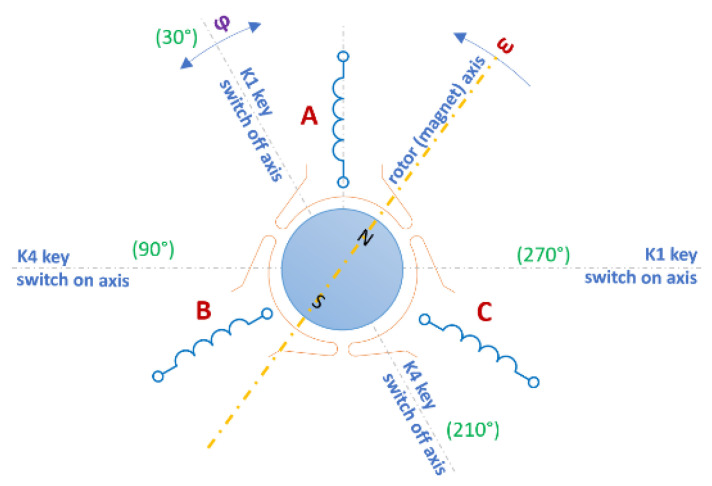
Motor windings with marked axes of switching on one of the bands. Changing the angle φ enables adjustment of the control mode and affects the torque characteristics. In the calculations, the boundary switching point of bands was assumed for such a rotor position in which the momentum is zero.

**Figure 4:**
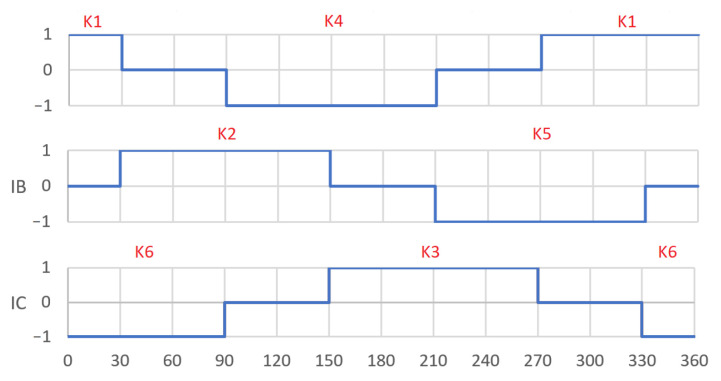
Adopted power supply system for successive coils.

The analyzed motors have a three-lane concentrated winding. Micromotors with a three-band winding are often used in household appliances. The construction of such motors is magnetically identical to a DC motor with three commutator divisions. Commutation consists of replacing the commutator and brushes with an electronic system. The figures below show the diagrams of the windings with some keys switching on the individual bands (Figure 2) and with the marked axes (Figure 3) for switching “on” and “off” the A band [25]. Thanks to the appropriate switching of the keys, a sequence of switching on successive coils can be obtained (Figure 4). The system assumed the angle of 0 degrees as consistent with the A axis.

In COMSOL Multiphysics^®^ software, using geometry parts created by the user can simplify the creation of complex geometries for simulation. The possibility of parameterizing individual dimensions and the possibility of using the “if” clause (if … else if … end if) increase the prototyping process.

**Figure 5:**
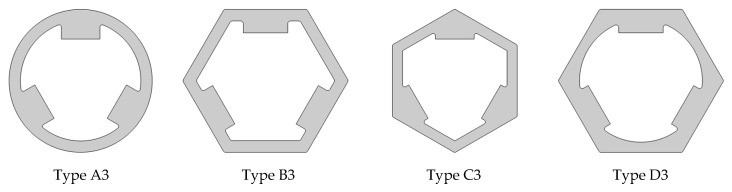
Stators (outer geometry parts).

**Figure 6:**
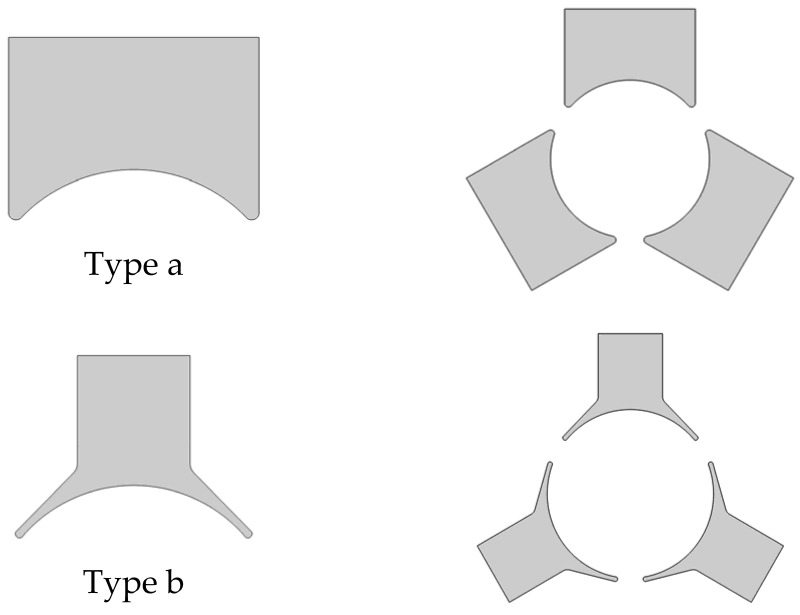
Stators (poles and inside geometry parts).

**Figure 7:**
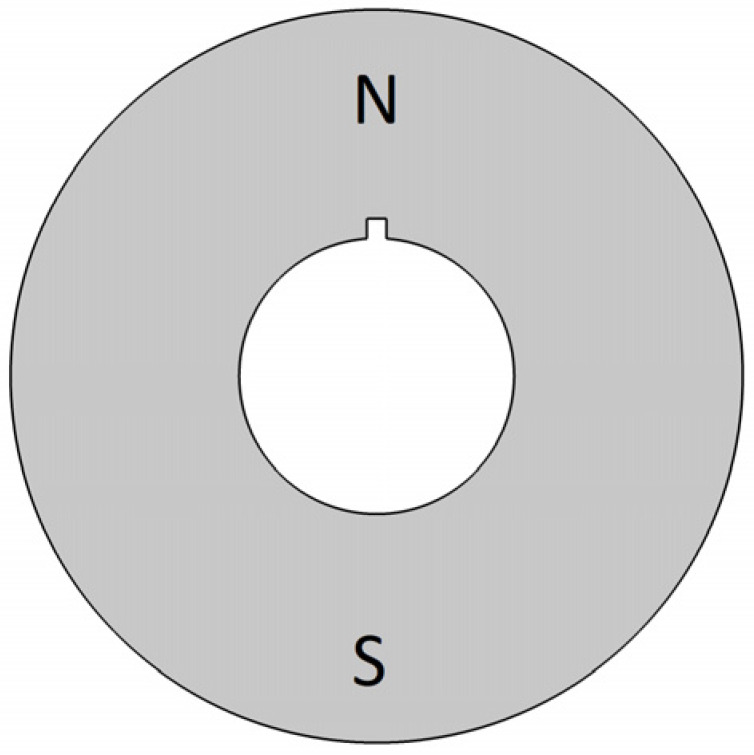
Rotor (only one type).

**Figure 8:**
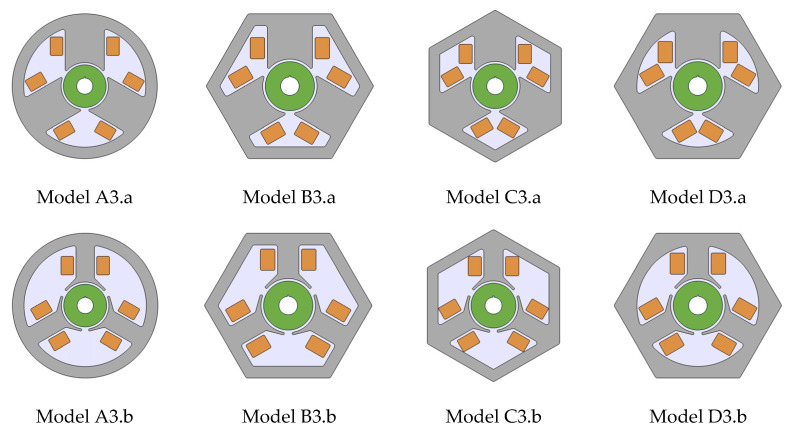
Analyzed configurations of the actuators/motors.

In this work, we used the software capabilities for quick comparative BLDC analysis that could be operated by either the actuator or motor. The individual variants of the device were realized as geometrical parts (Figure 5, Figure 6 and Figure 7), and the motors themselves were assembled as shown in Figure 8. The ring-shaped stator (A3) is the traditional solution. Another hexagonal-shaped stator (B3) corresponds to the reference motor, which is often used in household appliances. The next analyzed stator shapes (C3 and D3) can be found in the literature, for example, in Reference [26].

For each stator shape, two-pole solutions were adopted (Figure 6), simple poles (a) and poles with clearly wider pole pieces (b). The shape of the wide pole piece corresponds to the shape of the reference motor pole piece. In both cases (a and b), the same maximum width of the poles was kept.

In the case of the rotor (Figure 7), it is the simplest solution in the form of a uniform cylinder made of a two-pole magnet. It is a solution compatible with the motor that was the starting point for the analysis (Figure 1). Such rotors, together with their in-depth analysis, can be found in many publications. They can act as a rotor in traditional motors [27] or work in Active Magnetic Bearing (AMB) systems [28,29].

**Figure 9:**
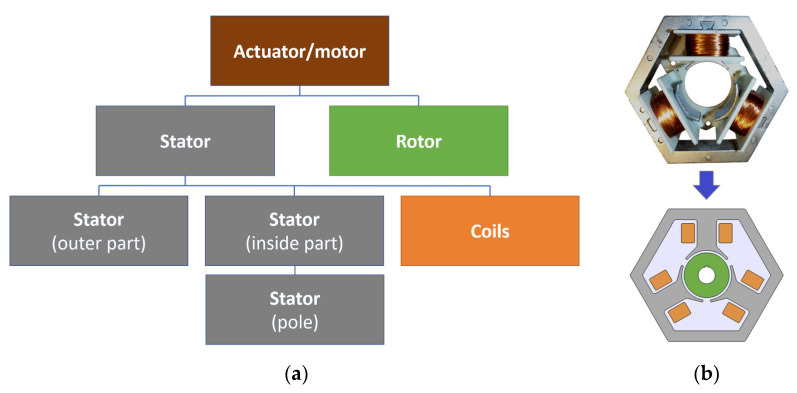
The idea of COMSOL parts and construction of motors: (**a**) diagrams and (**b**) model B3.b.

Figure 9 shows the idea of COMSOL parts, the construction of devices and an example of model realization based on an existing device.

**Figure 10:**
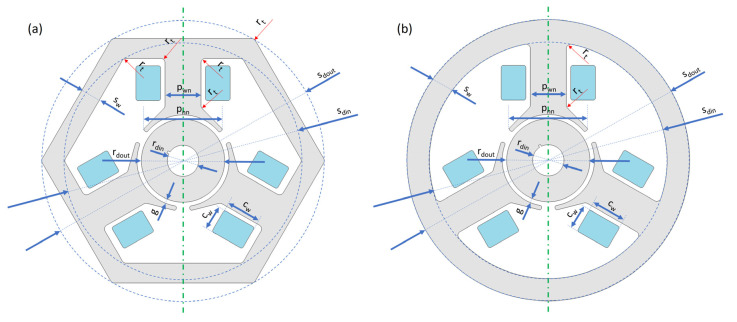
Analogies between the reference model, (**a**) B3.b, and the model (**b**) A3.a. For models from the _.b group, width p_wn_ = p_nn_.

The basic model is the model marked as B3.b3 (Figure 9), for which dimensions were adopted by following the dimensions of the real motor from Figure 1. For the remaining models, analogous dimensions were adopted. For example, motors with a circular stator have a stator radius corresponding to a wheel into which can be inscribed a hexagon corresponding to the reference motor. The values of physical dimensions (for example, stator outer/inner diameters, air gap length, rotor outer/inner diameters and motor width, and coil width/height) and electric parameters (for example, voltage and power) of the reference motor are reported in Table 1. The comparison of geometric dimensions for two different models is shown in Figure 10.

Two of the important features of motors, especially in household appliances, are the external dimensions and the external shape that determines the method of attachment. Motors with a hexagonal stator can be placed easily and stably on one side. Motors with a round, traditional stator take up more space compared to motors with a hexagonal stator. For the analyzed motors, the stator surface area was also compared, which directly translates into the mass of the motor. The results are presented in Table 2. It is worth noting that stators with narrow poles (marked as _.b) are of course lighter, but in this group, the reference motor is the best. Motors with the smallest and the largest stator surface are marked are colored gray; they are the lightest and heaviest devices, respectively.

## 4. Results

**Figure 11:**
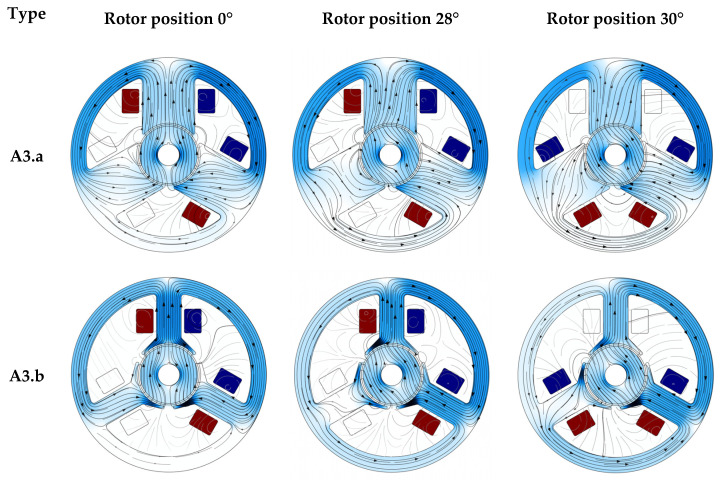
Examples: magnetic flux density and magnetic vector potential (lines). The position of the rotor in the subsequent drawings is 0, 28 and 30 degrees after the change of power bands, models A3.a and A3.b.

**Figure 12:**
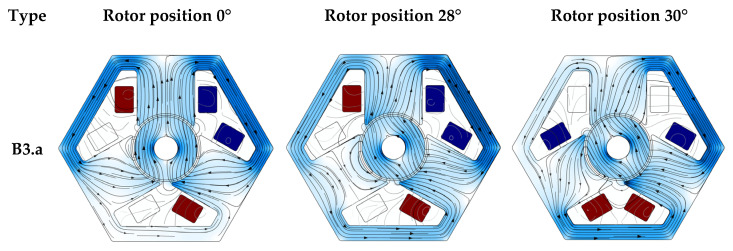

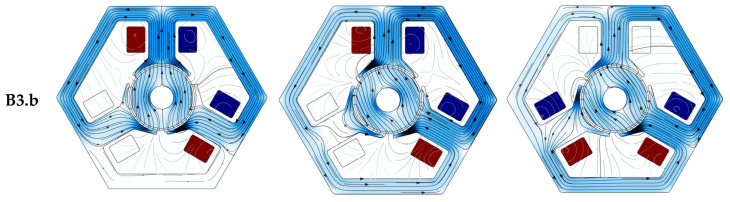
Examples: magnetic flux density and magnetic vector potential (lines). The position of the rotor in the subsequent drawings is 0, 28 and 30 degrees after the change of power bands, models B3.a and B3.b.

Examples of comparisons of different types of motors are presented in the figures below (Figure 11 and Figure 12). The figures show the distribution of the magnetic field in three cases. The first item applies to the case when the magnetic axis of the rotor is in front of one of the poles of the stator. The other two items show the situation before and after switching the voltage between the bands.

The figures clearly show which parts of the stator are characterized by an increased value of the magnetic flux density. It is also worth paying attention to the difference between wide and narrow poles. In the same rotor positions and with the same supply, it can be seen that the magnetic flux lines run in different paths.

**Figure 13:**
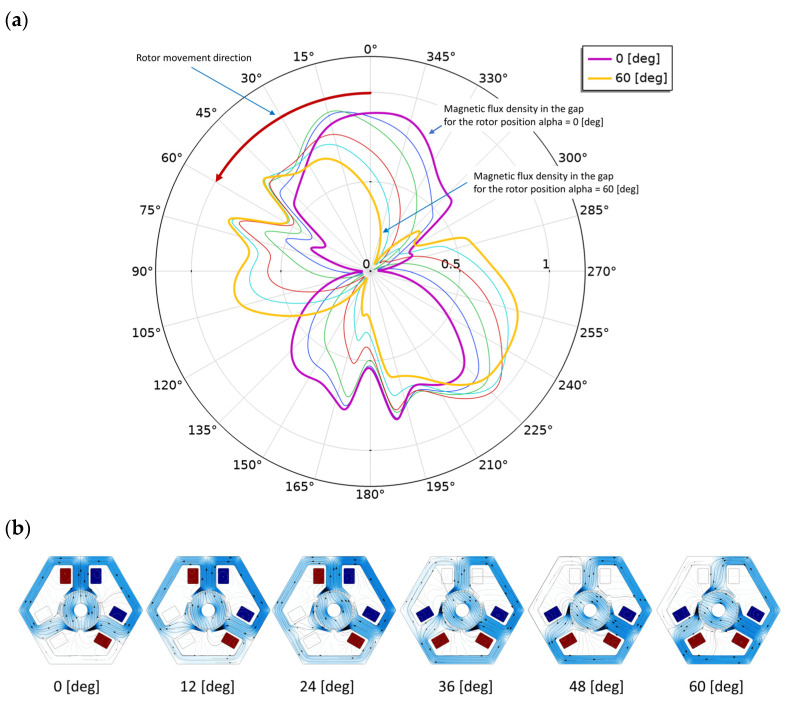
(**a**) Magnetic flux density in the gap for the rotor position from 0° to 60° for model B3.b. The following lines represent the rotor positions: 0° (purple), 12° (blue), 24° (green), 36° (red), 48° (turquoise) and 60° (yellow). Switching the coil voltage between 24° and 36° is considered. The radius corresponds to the magnetic flux density value in tesla. (**b**) Magnetic flux density and magnetic vector potential (lines) in successive rotor positions (rotated ever 12 degrees).

The next figure (Figure 13a) shows the method of visualization and analysis of the distribution of magnetic flux density in the gap in successive positions of the rotor. The rotor was rotated 12 degrees each time. Figure 13b shows successive positions of the rotor, together with magnetic flux density and magnetic vector potential (lines). These positions correspond to the lines in Figure 13a. As can be seen, this method of visualization allows for a good presentation of the phenomena occurring during the rotation of the rotor in one diagram. It shows characteristic places in the structure of the actuator and specific moments in time—it shows the moment of switching bands (between the green line (24 [deg]) and the red line (36 [deg])).

**Figure 14:**
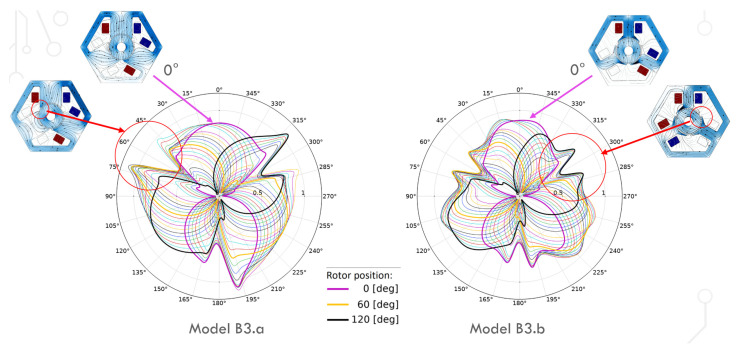
Magnetic flux density in the gap for the rotor position from 0° to 120° (rotated every 6 degrees), model B3.a and model B3.b. The switching of the coil voltage is considered. The radius corresponds to the magnetic flux density value in tesla. The line shapes are very similar in particular pairs of models (_.a and _.b), so only models B3.a and B3.b are presented.

The next figure (Figure 14) shows the distribution of the magnetic flux density in the gap in successive rotor positions (rotated every 6 degrees). For straight poles, maximum values (greater than 1 T) can be observed near one vertex of each pole. For models with a wider pole piece, in such rotor positions, the thin pole piece became magnetically saturated, so no such increase in the magnetic flux density was observed. This saturation can be observed in Figure 11 and Figure 12 for the rotation of the rotor by 28 and 30 degrees. The characteristic indentation in the magnetic flux density values for both models close to 60, 180 and 300 degrees (related to the model geometry) correspond to the gap between the poles.

**Figure 15:**
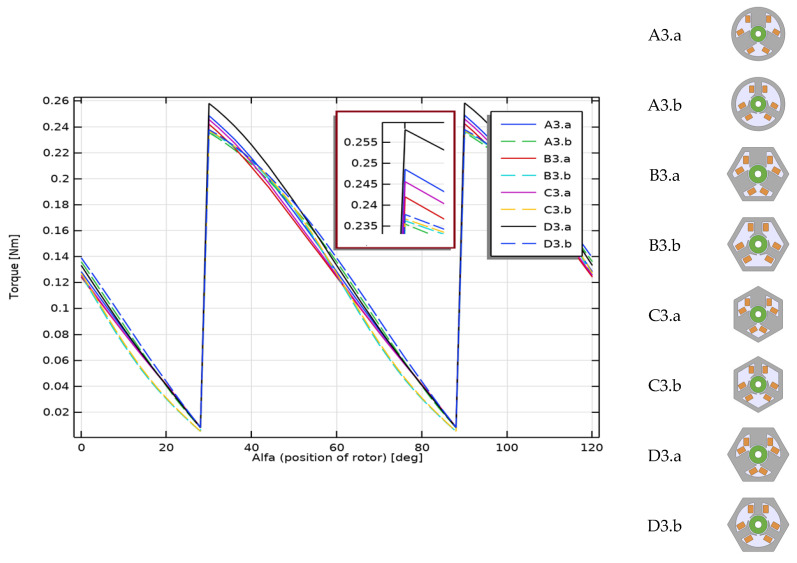
Axial torque for the rotor position from 0° to 120°. A band switching point was adopted for the rotor position, for which the torque is zero.

Figure 15 shows the results of calculations of the axial torque achieved by the analyzed motors during their work. You can see clearly, in the chart below, the group of wide-toothed motors (* .a, solid lines in the graph) that achieves the highest torques. The lowest values are observed for models B3.b (reference motor) and A3.b.

**Figure 16:**
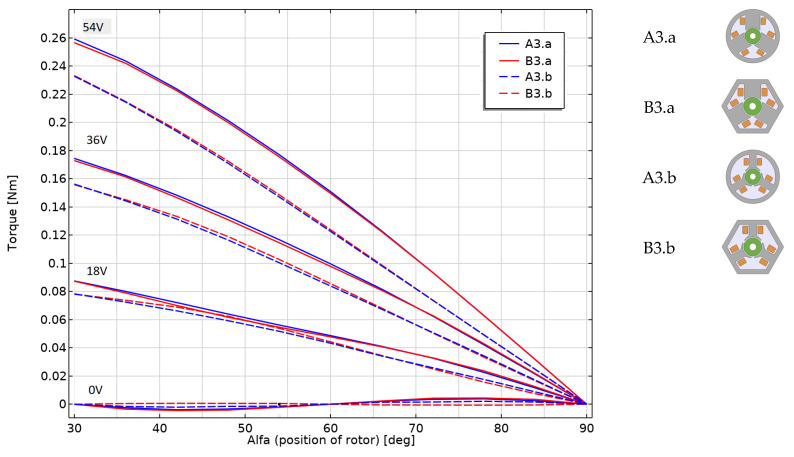
Axial torque for various models from the A3._ and B3._ group calculated for the angular position of the rotor from 30° to 90° for different supply voltages.

For a better comparison, the models were calculated for different supply voltages (set values of 0, 18 and 36 V; and the reference value was 54 V) and for different rotor positions, from the maximum torque position to the zero torque. In the adopted frame of reference, the range of angle changes is from 30° to 90°. Two coils connected in a series are powered simultaneously, so the voltage associated with one coil is half of the indicated value. Figure 16 compares the models marked A3 with the models marked B3, including the reference model, B3.b.

The presented characteristics for individual groups of models (_.a and _.b) and for a given voltage are similar. In these groups, the type of stator changes, while maintaining similar poles. On the other hand, you can clearly see the difference between the two types of poles for a specific stator. For the voltage of 54 V and the maximum torque, the difference is over 10%. For lower voltages, as well as for lower driving torques, the influence of the cogging torque is clearly visible.

Similar analyses were performed for the remaining groups of models (groups C._ and D._). The characteristics of the models from group C._ practically coincide with the models from group B._, while, in the characteristics of group D._, it was possible to notice a slightly greater difference in driving torque compared to the models from group B._. As you can see in Figure 16, for the straight pole, the driving torque was higher by a few percentage points, and for the tapered pole by a few percentage points less.

In the next step, the cogging torque was analyzed. There is a cogging torque between the stator teeth and the permanent magnet of the rotor, which depends on the position of the rotor. Reducing the cogging torque is very important to improve the efficiency of electric motors [30], especially for the PM BLDC [31]. A detailed description of the cogging torque limitation methods can be found in the literature (e.g., References [32,33,34,35]). Unfortunately, with such a small size and the rotor in the form of a single magnet, some of these methods—for example, the use of an axial tilt of the rotor by certain discrete steps—are impractical or even not applicable. Changing the shape of the poles is the best solution here.

**Figure 17:**
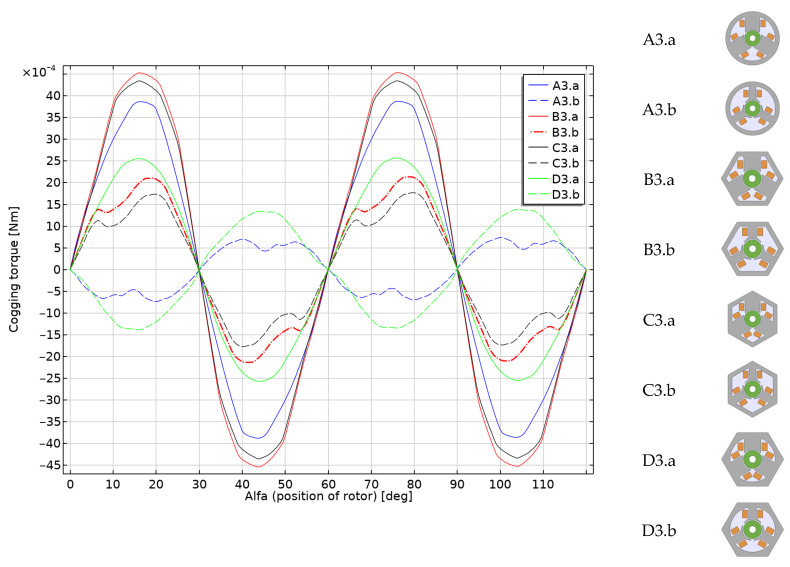
Cogging torque (Nm) for the rotor position from 0° to 120° for all models.

Figure 17 shows the results of the cogging torque calculations for individual devices. The highest values of cogging torque are observed in motors with wide poles (models B3.a, C3.a and A3.a). They are occurring at the level of 10% to 15% of the maximum torque achieved by these devices. The D3.a, B3.b and C3.b models are medium torque solutions. The lowest cogging torque is observed for the reference actuator B3.b (below 3%), and, similar to the actuator D3.b, it has the opposite sign compared to the other solutions.

## 5. Conclusions

This article presented a comparison of the existing sensorless PM BLDC actuator/motor used in household appliances with other design solutions with similar parameters. Such a broad comparison (for eight different models) was made possible by the use of COMSOL parts. COMSOL makes it easier to generate and compare solutions for different devices and systems. It takes less than a minute to create a single model. Full parameterization enables the construction of models with different dimensions and parameters.

The comparison was based on analyses of electromagnetic field distributions and calculated waveforms with parameters that are characteristic of the actuators. The reference motor has very good performance compared to other models. This actuator is the lightest of all the analyzed motors, and, therefore, the least material is used for its production. Its hexagonal structure is easy to assemble. The torque during work is slightly lower than in the other solutions; the cogging torque is lower than that motors with wide poles (_.a) but greater than other solutions with narrow poles (_.b).

According to the authors, an interesting approach is the implementation of diagrams showing the distribution of the magnetic induction in the gap in successive rotor positions with a slight angular shift and considering the switching of bands. The generation of these graphs, although long-lasting, enables a detailed analysis of the successive phases of motion concerning changes in the magnetic field. The comparison of the two models shows the differences related to the widths of the poles, as well as the gaps between the poles. The areas of higher magnetic saturation and the corresponding phases of motion are also clearly visible. A graph of this type can replace several or a dozen or so graphs of magnetic field distributions for successive rotor positions. This visualization method can be used for all other magnetic rotary actuators.

Since all the presented models are fully parameterized, it is also possible to optimize the presented structures. According to the authors, this should be the next stage of work with the prepared computing system.

## Figures and Tables

**Table 1:** Basic parameters of reference device.

Parameters	Description	Value
s_dout_	Stator outer diameter ^1^	63 mm
s_din_	Stator inner diameter ^1^	53 mm
s_w_	External stator width	5 mm
p_wn_	The width of the narrow pole	8 mm
p_ww_	The width of the wide pole	17 mm
g	Air gap length	1 mm
r_dout_	Rotor outer diameter	7 mm
r_din_	Rotor inner diameter	4 mm
m_w_	Motor width	19 mm
r_t_	Technical roundings	1 mm
c_w_	Coil width	5.5 mm
c_h_	Coin height	7.8 mm
R_c_	One coil resistance	39.6 ohm
U	Voltage	54 V
P	Power	30 W
v	Speed	3300 r/min

^1^ For hexagonal stators, this applies to the distance between opposite vertices of the hexagon.

**Table 2:** Comparison of the stators area.

Model	Stator Area (m^2^)
A3.a	1.78 × 10^−3^
A3.b	1.35 × 10^−3^
B3.a	1.49 × 10^−3^
B3.b	1.14 × 10^−3^
C3.a	1.55 × 10^−3^
C3.b	1.20 × 10^−3^
D3.a	1.63 × 10^−3^
D3.b	1.30 × 10^−3^

## Data Availability

Not applicable.

## References

[1] Petkovska L., Cvetkovski G. (2009). Hybrid analytical-FEM analysis of single phase Permanent Magnet Synchronous Motor. IEEE EUROCON.

[2] Novak L., Širok B., Hočevar M., Gatarić P. (2021). Influence of load mass and drum speed on fabric motion and performance of a heat pump tumble dryer. Dry. Technol..

[3] Di Barba P., Mognaschi M.E., Przybylski M., Rezaei N., Slusarek B., Wiak S. (2018). Field-based analysis and optimal shape synthesis of switched reluctance motors. Lect. Notes Electr. Eng..

[4] Nam H., Park S.B., Kang G.H., Hong J.P., Eom J.B., Jung T.U. (2004). Design to improve starting performance of line-start synchronous reluctance motor for household appliances. Conference Record of the 2004 IEEE Industry Applications Conference, Proceedings of the 39th IAS Annual Meeting, Seattle, WA, USA, 3–7 October 2004.

[5] Saghin S.M., Ghaheri A., Shirzad H., Afjei E. (2021). Performance optimisation of a segmented outer rotor flux switching permanent magnet motor for direct drive washing machine application. IET Electr. Power Appl..

[6] Mutluer M. (2021). Analysis and design optimization of permanent magnet motor with external rotor for direct driven mixer. J. Electr. Eng. Technol..

[7] Abdeljawed H.B., El Amraoui L. (2022). Simulation and rapid control prototyping of DC powered universal motors speed control: Towards an efficient operation in future DC homes. Int. J. Eng. Sci. Technol..

[8] Degano M., Murataliyev M., Shuo W., Barater D., Buticchi G., Jara W., Bianchi N., Galea M., Gerada C. (2021). Optimised Design of Permanent Magnet Assisted Synchronous Reluctance Machines for Household Appliances. IEEE Trans. Energy Convers..

[9] Arunkumar S., Sundaram N.M., Thottungal R., Shreya A. A Bridge Type DC-DC Converter fed BLDC Motor Drive for Household Appliances. Proceedings of the International Conference on Advancements in Electrical, Electronics, Communication, Computing and Automation (ICAECA).

[10] Xu D., Wang B., Zhang G., Wang G., Yu Y. (2018). A review of sensorless control methods for AC motor drives. CES Trans. Electr. Mach. Syst..

[11] Hembach H., Evans S.A., Gerling D. Systematic comparison of BLDC motors for small automotive water pump applications. Proceedings of the 18th International Conference on Electrical Machines.

[12] Lee W.J., Sul S.K. (2006). A new starting method of BLDC motors without position sensor. IEEE Trans. Ind. Appl..

[13] Jeong C.L., Hur J. (2016). A novel proposal to improve reliability of spoke-type BLDC motor using ferrite permanent magnet. IEEE Trans. Ind. Appl..

[14] Krykowski K., Hetmańczyk J., Gałuszkiewicz Z., Miksiewicz R. (2011). Computer analysis of high-speed PM BLDC motor properties. COMPEL-Int. J. Comput. Math. Electr. Electron. Eng..

[15] Misal S.R., Bhasme N.R. A review of multi-switch BLDC motor drive. Proceedings of the 2017 Innovations in Power and Advanced Computing Technologies (i-PACT).

[16] Sun X., Li Z., Wang X., Li C. (2020). Technology Development of Electric Vehicles: A Review. Energies.

[17] Kim S.C., Sangam N., Pagidipala S., Salkuti S.R., Salkuti S.R., Ray P. (2022). Design and Analysis of BLDC Motor Driver for Hybrid Electric Vehicles. Next Generation Smart Grids: Modeling, Control and Optimization.

[18] Vidhya H., Allirani S. A Literature Review on Electric Vehicles: Architecture, Electrical Machines for Power Train, Converter Topologies and Control Techniques. Proceedings of the 2021 International Conference on Computational Performance Evaluation (ComPE).

[19] Kumar D., Gupta R.A. (2021). A comprehensive review on BLDC motor and its control techniques. Int. J. Power Electron..

[20] Tejaswini K., Qutubuddin M.D. (2021). Simulation And Design of Novel System for BLDC Motor Using Advanced Drive System Converter Circuit. J. Eng. Sci..

[21] Stănică D.M., Bizon N., Arva M.C. A brief review of sensorless motors position control. Proceedings of the 2021 13th International Conference on Electronics, Computers and Artificial Intelligence (ECAI).

[22] Gamazo-Real J.C., Vázquez-Sánchez E., Gómez-Gil J. (2010). Position and speed control of brushless DC motors using sensorless techniques and application trends. Sensors.

[23] Mohan H., Pathak M.K., Dwivedi S.K. (2020). Sensorless control of electric drives—A technological review. IETE Tech. Rev..

[24] Wang G., Valla M., Solsona J. (2019). Position sensorless permanent magnet synchronous machine drives—A review. IEEE Trans. Ind. Electron..

[25] Glinka T. (2008). Electric motors with permanent magnets. Przegląd Elektrotechniczny.

[26] Hong J.P., Ha K.H., Lee J. (2002). Stator pole and yoke design for vibration reduction of switched reluctance motor. IEEE Trans. Magn..

[27] Xue S., Xu H., Fang C. The effect of stator slot and air gap length on high speed brushless PM motor. Proceedings of the 7th International Power Electronics and Motion Control Conference.

[28] Kiani M., Salarieh H., Alasty A., Darbandi S.M. (2016). Stabilization of a Three-Pole Active Magnetic Bearing by Hybrid Control Method in Static Mode. Int. J. Mech. Mechatron. Eng..

[29] Zhang W., Zhu H. (2017). Radial magnetic bearings: An overview. Results Phys..

[30] Hiremath R. Finite element study of induced Emf, cogging torque and its reductions in BLDC motor. Proceedings of the 2017 International Conference on Intelligent Computing, Instrumentation and Control Technologies, ICICICT.

[31] Yaz M., Cetin E. (2021). Brushless Direct Current Motor Design and Analysis. COJ Electron. Commun..

[32] Zhu L., Jiang S.Z., Zhu Z.Q., Chan C.C. (2009). Analytical Methods for Minimizing Cogging Torque in Permanent-Magnet Machines. IEEE Trans. Magn..

[33] Anuja T.A., Doss M.A.N. (2021). Reduction of Cogging Torque in Surface Mounted Permanent Magnet Brushless DC Motor by Adapting Rotor Magnetic Displacement. Energies.

[34] Anuja T.A., Doss M.A.N. (2022). Asymmetrical Magnets in Rotor Structure of a Permanent Magnet Brushless DC Motor for Cogging Torque Minimization. J. Electr. Eng. Technol..

[35] Li Z., Yu X., Wang X., Xing X. (2021). Optimization and Analysis of Cogging Torque of Permanent Magnet Spherical Motor. IEEE Trans. Appl. Supercond..

